# The smell of lung disease: a review of the current status of electronic nose technology

**DOI:** 10.1186/s12931-021-01835-4

**Published:** 2021-09-17

**Authors:** I. G. van der Sar, N. Wijbenga, G. Nakshbandi, J. G. J. V. Aerts, O. C. Manintveld, M. S. Wijsenbeek, M. E. Hellemons, C. C. Moor

**Affiliations:** 1 Department of Respiratory Medicine, Erasmus MC, University Medical Center, Rotterdam, The Netherlands; 2 Department of Cardiology, Erasmus MC, University Medical Center, Rotterdam, The Netherlands

**Keywords:** Electronic nose, Breath analysis, Respiratory medicine, Personalised medicine, Machine learning, Sensor technology

## Abstract

There is a need for timely, accurate diagnosis, and personalised management in lung diseases. Exhaled breath reflects inflammatory and metabolic processes in the human body, especially in the lungs. The analysis of exhaled breath using electronic nose (eNose) technology has gained increasing attention in the past years. This technique has great potential to be used in clinical practice as a real-time non-invasive diagnostic tool, and for monitoring disease course and therapeutic effects. To date, multiple eNoses have been developed and evaluated in clinical studies across a wide spectrum of lung diseases, mainly for diagnostic purposes. Heterogeneity in study design, analysis techniques, and differences between eNose devices currently hamper generalization and comparison of study results. Moreover, many pilot studies have been performed, while validation and implementation studies are scarce. These studies are needed before implementation in clinical practice can be realised. This review summarises the technical aspects of available eNose devices and the available evidence for clinical application of eNose technology in different lung diseases. Furthermore, recommendations for future research to pave the way for clinical implementation of eNose technology are provided.

**Supplementary Information:**

The online version contains supplementary material available at 10.1186/s12931-021-01835-4.

## Background

The field of pulmonary medicine has rapidly evolved over the﻿ last decades, with increasing knowledge about pathophysiology and aetiology leading to better targeted treatment strategies. Nevertheless, many chronic lung diseases have non-specific, often overlapping symptoms, which delays the diagnostic process and timely start of adequate treatment. Moreover, even specific disease entities can be very heterogeneous with varying phenotypes, and thus disease courses and optimal treatment strategies vary per patient. Accurate, non-invasive, real-time diagnostic tools and biomarkers to predict disease course and response to therapy are currently lacking in most lung diseases, but are indispensable to achieve a personalised approach for individual patients.

An emerging tool that has the potential to meet this need is an electronic nose (eNose). This device ‘smells’ exhaled breath for clinical diagnostics, a concept probably as old as the field of medicine itself. Exhaled breath contains thousands of molecules, also known as volatile organic compounds (VOCs). These VOCs can be divided into compounds derived from the environment (exogenous VOCs) and compounds that are the result of biological processes in the body (endogenous VOCs). Endogenous VOCs can be associated with normal physiology, but also with pathophysiological inflammatory or metabolic activity [1, 2]. Identification of individual VOCs using techniques as gas chromatography or mass spectrometry is a specific but time-consuming exercise. An eNose can be used in real-time to recognise patterns of VOCs and has therefore potential as point-of-care tool in clinical practice.

The aim of this paper is to review the current clinical evidence on eNose technology in lung disease, regarding diagnosis, monitoring of disease course and therapy evaluation. In addition, technical aspects and available eNose devices are discussed.

## eNose technology

In the time of Hippocrates, it was already acknowledged that exhaled breath can provide information about health conditions [3]. For instance, a sweet acetone breath odour indicates diabetes, a fishy smell suggests liver disease, and wounds with smell of grapes point towards pseudomonas infections [4]. Initial breath analysis studies were performed using gas chromatography or mass spectrometry. Throughout the last decades, more techniques were developed for breath analysis, for example ion mobility spectrometry, selected ion flow tube mass spectrometry and laser spectrometry [5]. Although these techniques became more advanced during the years and are very precise in identifying individual VOCs, they are very complex, laborious and thus not suitable as a real-time clinical practice tool.

Exhaled breath analysis by use of eNose technology is recently gaining increasing attention. An eNose is defined as “an instrument which comprises of an array of electronic-chemical sensors with partial specificity and an appropriate pattern recognition system, capable of recognising simple or complex odours” [6]. Sensors are used in eNoses to generate a singular response pattern. The sensors can generally be divided into three categories: electrical, gravimetric, and optical sensors. Each type responds to analytes (i.e. VOCs) in a specific way, and all types have a high sensitivity. Each sensor has advantages and disadvantages, without one type being superior in general. Electrical sensors consist of an electronic circuit connected to sensory materials. Upon binding with specific analytes, an electrical response is provided [7–10]. Consequently, a variation in electrical property of the sensor surface can be detected. Electrical sensors are low-cost, but are sensitive to temperature changes and have a limited sensor life [11]. Gravimetric (or mass sensitive) sensors label analytes based on changes in mass, amplitude, frequency, phase, shape, size, or position. Gravimetric sensors contain a complex circuitry and are sensitive to humidity and temperature [11]. Finally, optical sensors detect a change in colour, light intensity or emission spectra upon analyte binding. Optical sensors are insensitive to environmental changes, but are the most technically complex sensor-array systems and are not portable due to breakable optics and components. Due to the high complexity, they are more expensive than the other sensor types [11]. For each type of sensor, a more in depth explanation can be found in the Additional file 1.

**Fig. 1:**
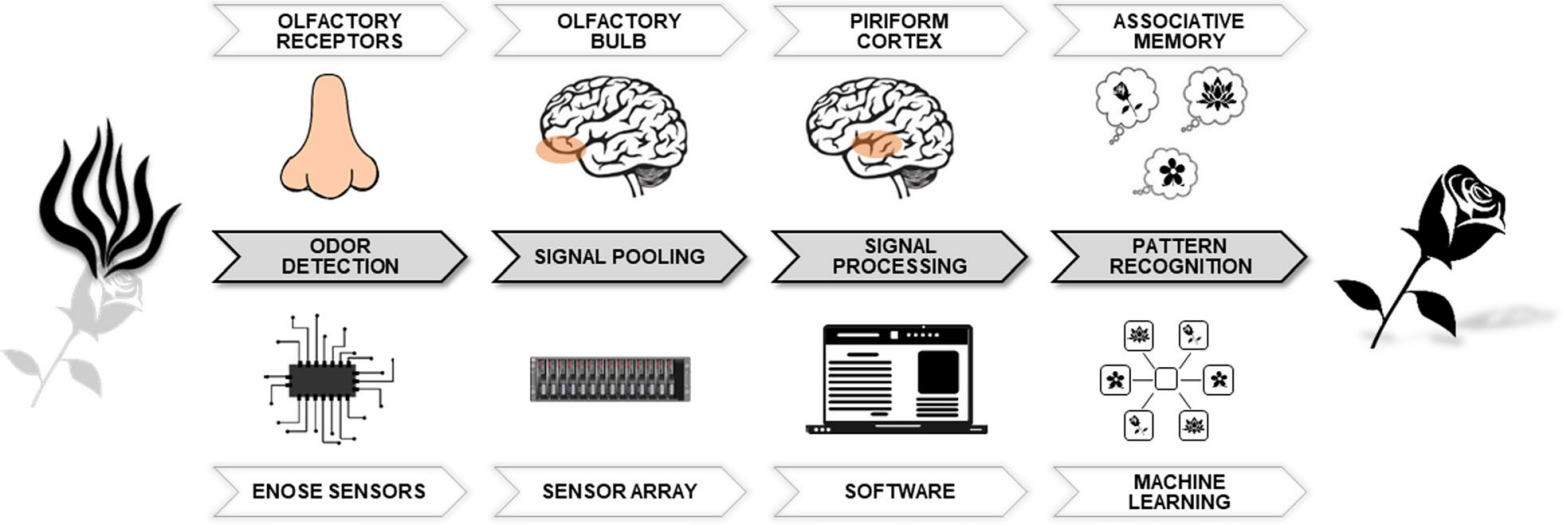
Schematic comparison of eNose technology and the olfactory system [12]

Detection and recognition of odours by an eNose is similar to the functioning of the mammalian olfactory system (Fig. 1). First, an odour is detected (by olfactory receptors in a human nose or eNose sensors), which sends off various signals (to the cortex or software). Then, these signals are pooled together and processed into a pattern. This pattern can be recognised as a particular smell (e.g. a flower) [12]. As a result, an eNose can differentiate between diseases by analysing and comparing the smelled ‘breathprints’ (i.e. VOC patterns) with those previously learned. The devices are hand-held, patient friendly, easy-to-use and feasible as point-of-care test.

### Analysis methods

To analyse eNose breathprints, pattern recognition by machine learning is most commonly used. A machine learning model uses algorithms which automatically improve due to experience with previously presented data. These models are in general established using a five step process: data collection, data preparation, model building, model evaluation, and model improvement. Machine learning is categorised into unsupervised, supervised, and reinforcement learning [13]. In supervised learning, the algorithms are trained with labelled data input, the desired output is thus known. On the contrary, unsupervised learning allows the algorithm to recognise patterns in the data, and groups data without providing labels. Lastly, reinforcement learning encompasses the training of the machine learning models to generate decision sequences. The latter is not used in the eNose studies reviewed in this paper.

Several machine learning models have been proposed as appropriate algorithms for modelling complex nonlinear relationships in medical research data, such as breathprints. These models include, amongst others, artificial neural networks (mimicking the structure of animal brains to model functions), ensemble neural networks (many neural networks working together to solve a problem), and support vector machines (SVM, creating﻿ a hyperplane which allows the modelling of highly complex relationships) [14, 15]. A comparison between eNose studies show that SVM algorithm is most frequently used (10 out of 17 studies in 2019) [15]. Possibly, this is due to the fact that this is the easiest model to use for researchers new to machine learning. Another factor can be the existence of many programming languages with well-supported libraries for SVM algorithms. SVM also possesses a high accuracy, is not very prone to overfitting, and is not overly influenced by noisy data [15]. Nonetheless, there is no consensus about the optimal model for breathprint analysis.

### Available eNoses

Various eNose devices have been developed and studied in different lung diseases. Table 1 provides an overview of the specifications of devices used in studies reviewed in this paper. The choice of an eNoses device may, among others, depend on the measurement setting. For example for the BIONOTE, Cyranose 320, PEN3, and Tor Vergata eNoses the exhaled breath is captured into sample bags or cartridges which makes it possible to collect on-site and store samples for later analyses. In other settings, it could be preferable that the eNose is easily portable, like the Aeonose. The SpiroNose is the only eNose that is capable of adjusting for disturbances from ambient air using its external sensors.

**Table 1:** Characteristics of available eNoses

	Aeonose	BIONOTE	Cyranose 320	PEN3	SpiroNose	Tor Vergata
Company	The eNose company, Zutphen, the Netherlands	Campus Bio-Medico University, Rome, Italy	Sensigent, California, United States (previously known as: Smith Detections)	Airsense Analytics GmbH, Schwerin, Germany	Breathomix, Leiden, the Netherlands (previously produced by: Comon Invent)	Tor Vergata University, Rome, Italy
Working Principle (i.e. sensors)	Electrical sensors	Gravimetric sensors	Electrical sensors	Electrical sensors	Electrical sensors	Gravimetric sensors
Sensing material	MOS	QCM	Conducting polymer	MOS	MOS	QCM
Array composition	1 array; 3 sensors	1 array; 7 sensors operating at 4 different temperatures	1 array; 32 different polymers	1 array; 10 different sensors	4 exhaled breath and 4 reference arrays; 7 different sensors per array	1 array; 8 sensors
Breath collection	Tidal breathing straight into eNose	Tidal breathing into Pneumopipe cartridge	Exhalation into sample bag	Exhalation into sample bag	Exhalation straight into eNose	Exhalation into sample bag
	NA	3 min tidal breathing	5 min tidal breaths, deep inhale, exhalation	5 min tidal breathing, deep in- and exhalation	5 tidal breaths, deep inhale, breath hold, slow exhalation	Deep in- and exhalation
Image						
Image source	www.enose.nl	Rocco et al. 2016 [16]	www.sensigent.com/products/cyranose.html	www.airsense.com/sites/default/files/flyer_pen.pdf	www.breathomix.com	Tor Vergata University

An overview of specifications of eNose devices used in studies reviewed in this paper. eNose prototypes are not included. *BIONOTE*  biosensor-based multisensorial system for mimicking nose tongue and eyes, *eNose*  electric nose, *MOS*  metal oxide semiconductor, *PEN*  portable electronic nose, *QCM*  quartz crystal microbalance. Images are used with approval of the eNose companies

**Fig. 2:**
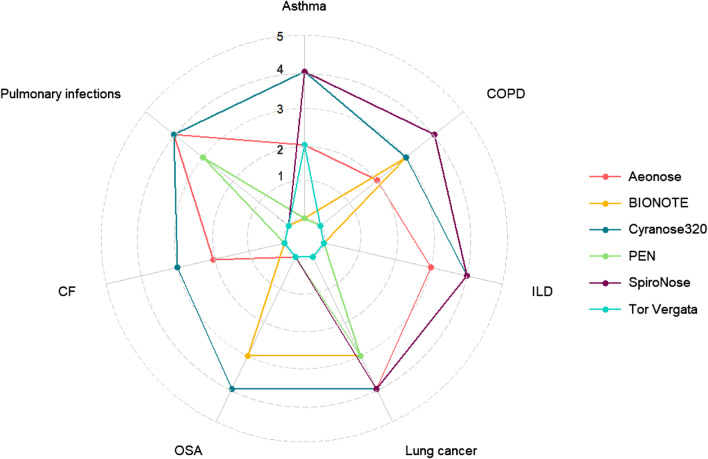
Radar plot of development stages per eNose and disease. Studies were divided into five different stages: (1) proof of concept study; (2) cohort size of diseased participants less than fifty; (3) cohort size of diseased participants equal or more than fifty; (4) study cohort with an external validation cohort; (5) evaluation of clinical implementation. The highest stage reached for each eNose per lung disease is displayed. eNose prototypes are not included. *BIONOTE*  biosensor-based multisensorial system for mimicking nose tongue and eyes, *CF*  cystic fibrosis, *COPD*  chronic obstructive pulmonary disease, *ILD*  interstitial lung disease, *OSA*  obstructive sleep apnoea, *PEN*  portable electronic nose.

The stage of development towards a clinically implemented tool differs substantially per device and disease. Before clinical implementation, each specific eNose has to be tested as a proof of concept and consecutively in substantial cohorts for each specific disease. Subsequently, data validation and clinical implementation needs to be assessed in real-life cohorts. To give more insights in the stage of development for each eNose per lung disease, we divided studies in five different stages: (1) proof of concept study; (2) cohort size of diseased participants less than fifty; (3) cohort size of diseased participants equal or more than fifty; (4) study cohort with an external validation cohort; (5) evaluation of clinical implementation. An overview of the progress per eNose and disease is visualised in Fig. 2. To the best of our knowledge, none of the devices are currently used in clinical pulmonology practice.

## Current clinical application

On 21 October 2020, a systematic literature search was performed in the databases Embase, Medline (Ovid), and﻿ Cochrane Central. Search terms and selection criteria are described in the Additional file 2. Table 2 provides an overview of design and results of all studies in this review.

**Table 2:** Literature overview eNose technology in lung disease

	﻿Study participants		Outcome measures	Results				eNose	Statistical breathprint analysis
***Asthma***									
Dragonieri, 2007 [18]	n = 20 asthma • ﻿n = 10 mild • n = 10 severe n = 20 HC • n = 10 old • n = 10 young		Diagnostic accuracy	Mild vs young HC CVV 100%	Severe vs old HC CVV 90%		Mild vs severe CVV 65%	Cyranose 320	PCA; CDA
Fens 2009 [19]	n = 20 asthma n = 30 COPD n = 20 non-smoking HC n = 20 smoking HC		Diagnostic accuracy	COPD vs asthma CVA 96%	COPD vs smoking HC CVA 66%		Non-smoking vs smoking HC Not significant	Cyranose 320	PCA
Lazar 2010 [20]	n = 10 asthma • induction of bronchoconstriction with methacholine or saline n = 10 controls		Disease course	Bronchoconstriction causes no significant change in breathprint				Cyranose 320	PCA; mixed model analysis
Montuschi 2010 [21]	n = 27 asthma n = 24 HC		Diagnostic accuracy	eNose only Acc 87.5%	eNose + FeNO Acc 95.8%			Tor Vergata	PCA; feed-forward neural network
Fens 2011 [26]	*Training:* [19] n = 20 asthma n = 20 COPD	*Validation*: n = 60 asthma • n = 21 fixed obstruction • n = 39 classic n = 40 COPD	Diagnostic accuracy	*Validation*: Classic asthma vs COPD Sens 85% Spec 90% AUC 0.93 (0.84–1.00) Acc 83%	*Validation*: Fixed asthma vs COPD Sens 91% Spec 90% AUC 0.95 (0.87–1.00) Acc 88%		*Validation*: Fixed vs classic asthma No significant difference	Cyranose 320	PCA; CDA
Van der Schee 2013 [22]	n = 25 asthma n = 20 HC		Diagnostic accuracy	Before OCS Sens 80.0% Spec 65.0% AUC 0.766 ± 0.14	After OCS Sens 84.0% Spec 80% AUC 0.862 ± 0.12		Before OCS (FeNO only) AUC 0.738 ± 0.15	Cyranose 320	PCA; CDA
	n = 18 asthma • maintenance ICS, stop ICS (4 weeks) and OCS (2 weeks)		Therapeutic effect	OCS responsive vs not Sens 90.9% Spec 71.4% AUC 0.883 (± 0.16)					
	n = 25 asthma • maintenance ICS, stop ICS (4 weeks) and OCS (2 weeks) • n = 13 Loss of control (LOC)		Disease course	LOC vs no LOC Sens 90.9% Spec 71.4% AUC 0.814 ± 0.17	Correlation sputum eos—breathprint R = 0.601				
Plaza 2015 [30]	n = 24 eosinophilic asthma n = 10 neutrophilic asthma n = 18 paucigranulocytic asthma		Diagnostic accuracy	Neutro vs pauci Sens 94% Spec 80% AUC 0.88 CVA 89%	EoS vs neutro Sens 60% Spec 79% AUC 0.92 CVA 73%		EoS vs pauci Sens 55% Spec 87% AUC 0.79 CVA 74%	Cyranose 320	PCA; CDA
Brinkman 2017 [32]	n = 22 asthma, induced LOC • maintenance ICS, stop ICS (8 weeks) and restart ICS		Disease course	Baseline vs LOC Acc 95%	LOC vs recovery Acc 86%		Correlation sputum eos—breathprint Not significant	Cyranose 320	PCA
Bannier 2019 [23]	n = 20 asthma (age > 6 years) n = 22 HC		Diagnostic accuracy	Sens 74% Spec 74% AUC 0.79				Aeonose	ANN
Brinkman 2019 [31]	n = 78 severe asthma • n = 51 longitudinal follow-up		Clustering	3 clusters (baseline), acc 93% Diff﻿erences: chronic OCS use, percent serum eosinophil and neutrophil count			Follow-up (18 months) n = 21 cluster stable n = 30 migrated	Cyranose 320, Tor Vergata, Comon Invent	PCA; Ward clustering; Non-hierarchical K-means clustering; PLS-DA; PAM; Topological data analysis
Cavaleiro Rufo 2019 [34]	n = 64 suspected asthma (age 6–18 years) • n = 45 asthma • n = 29 persistent • n = 16 intermittent • n = 19 no asthma		Diagnostic accuracy	Asthma vs no asthma Sens 77.8% Spec 84.2% AUC 0.81 (0.69–0.93) Acc 79.7%	Persistent vs no asthma Sens 79.7% Spec 68.6% AUC 0.81 (0.70–0.92) Acc 79.7%		Intermittent vs no asthma Not significant	Cyranose 320	PCA; Hierarchical clustering
Dragonieri 2019 [24]	*Training:* n = 14 AAR n = 14 rhinitis n = 14 HC	*Validation*: n = 7 AAR n = 7 rhinitis n = 7 HC	Diagnostic accuracy	*Training:* AAR vs HC AUC 0.87 (0.70–0.97) CVA 75.0%	*Validation*: AAR vs HC AUC 0.77 (0.62–0.93) CVA 67.4%		*Validation*: AAR vs rhinitis AUC 0.92 (0.84–1.00) CVA 83.1%	Cyranose 320	PCA; CDA
Abdel-Aziz 2020 [118]	*Training:* n = 486 atopic asthma (age > 4 years)	*Validation*: n = 169 atopic asthma (age > 4 years)	Diagnostic accuracy	*Training:* AUC 0.837–0.990 Sens, spec and acc only visually available	*Validation*: AUC 0.18–0.926 Sens, spec and acc only visually available			Cyranose 320, Tor Vergata, Comon Invent, SpiroNose	PLS-DA; adaptive least absolute shrinkage and selection operator; gradient boosting machine
Farraia 2020 [28]	*Training:* n = 121 asthma suspected (age > 6 years)	*Validation*: n = 78 asthma suspected (age > 6 years)	Clustering	*Training:* 3 clusters (hierarchic), differences: food/drink intake 2 h prior to sampling, percentage of asthma diagnosis in group, PEF%, age < 12 y			*Validation*: 3 clusters (hierarchic), differences: food/drink intake 2 h prior to sampling	Cyranose 320	Unsupervised hierarchic clustering; Non-hierarchical K-means clustering; PAM
Tenero 2020 [25]	n = 28 asthma (age 6–16 years) • n = 9 controlled • n = 7 partially controlled • n = 12 uncontrolled n = 10 HC		Diagnostic accuracy	HC + controlled vs. partially + uncontrolled Sens 79% Spec 84% AUC 0.85 (0.72–0.98)				Cyranose 320	Penalized logistic regression PCA
***Chronic obstructive pulmonary disease (COPD)***									
Fens 2011 [45]	n = 28 GOLD I + II • airway inflammation (sputum eosinophil cationic protein and myeloperoxidase)		Disease course	Correlation eosinophil cationic protein and breathprint r = 0.37	Correlation myeloperoxidase and breathprint Not significant		Airway inflammation vs no Sens 50–73% Spec 77–91% AUC 0.66–0.86	Cyranose 320	PCA
Hattesohl 2011 [37]	n = 23 COPD (pure exhaled breath, PEB) n = 10 COPD (exhaled breath condensate, EBC) n = 10 HC (EBC, PEB) n = 10 AATd (EBC, PEB)		Diagnostic accuracy	COPD vs HC Sens 100% Spec 100% CVV PEB 67.6% CVV EBC 80.5%	COPD vs AATd Sens 100% Spec 100% CVV PEB 58.3% CVV EBC 82.0%		HC vs AATd Sens 100% Spec 100% CVV PEB 62.0% CVV EBC 59.5%	Cyranose 320	LDA
	n = 11 AATd COPD (PEB) • augmentation therapy		Therapeutic effect	Before vs 6 d after therapy Sens 100% Spec 100% CVV 53.3%					
Fens 2013 [42]	n = 157 COPD		Clustering	4 clusters (acc 97.4%) Differences: airflow limitation, health related QoL, sputum production, dyspnoea, smoking history, co-morbidity, radiologic density, gender				Cyranose 320	Hierarchical cluster analysis Non-hierarchical K-means clustering
Sibila 2014 [41]	n = 10 COPD bacterial colonised n = 27 COPD non-colonised n = 13 HC		Diagnostic accuracy	Colonised vs non-colonised Sens 82% Spec 96% AUC 0.922 CVA 89%	HC vs non-colonised Sens 81% Spec 86% AUC 0.937 CVA 83%		HC vs colonised Sens 80% Spec 93% AUC 0.986 CVA 87%	Cyranose 320	PCA; CDA
Cazzola 2015 [38]	n = 27 COPD • n = 8 AECOPD ≥ 2 per year • n = 19 AECOPD < 2 per year n = 7 HC		Diagnostic accuracy	COPD vs HC Sens 96% Spec 71% CVA 91%	AECOPD ≥ 2 vs < 2 per y Not significant			Prototype (6 QMB sensors)	PLS-DA
Shafiek 2015 [39]	n = 50 COPD • n = 17 sputum PPM growth n = 93 AECOPD • n = 42 sputum PPM growth n = 30 HC		Diagnostic accuracy	COPD vs HC Sens 70–72% Spec 70–73%	COPD vs AECOPD no PPM Sens 89% Spec 48% (with PPM not significant)		AECOPD PPM vs AECOPD no PPM Sens 88% Spec 60%	Cyranose 320	LDA; SLR
	n = 61 AECOPD • during and 2 months after recovery		Disease course	During vs recovery Sens 74% Spec 67%					
Van Geffen 2016 [46]	n = 43 AECOPD • n = 18 with viral infection • n = 22 with bacterial infection		Diagnostic accuracy	With vs without viral infection Sens 83% Spec 72% AUC 0.74	With vs without bacterial infection Sens 73% Spec 76% AUC 0.72			Aeonose	ANN
De Vries 2018 [43]	*Training:* n = 321 asthma/COPD	*Validation*: n = 114 asthma/COPD	Clustering	5 clusters Differences: ethnicity, systemic eosinophilia/ neutrophilia, FeNO, BMI, atopy, exacerbation rate				SpiroNose	PCA; Unsupervised Hierarchical clustering
Finamore 2018 [49]	n = 63 COPD • n = 32 n6MWD worsened 1 year • n = 31 n6MWD stable or improved 1 year		Disease course	n6MWD change predicted by eNose Sens 84% Spec 88% CVA 86%	n6MWD change predicted by eNose + GOLD Sens 81% Spec 78% CVA 79%			BIONOTE	PLS-DA
Montuschi 2018 [50]	n = 14 COPD • maintenance ICS, stop ICS (4 weeks) and restart ICS		Therapeutic effect	Maintenance vs restart ICS Change in 15 of 32 Cyranose sensors; 3 of 8 Tor Vergata sensors	Maintenance vs restart ICS Spirometry + breathprint prediction model AUC 0.857			Cyranose 320, Tor Vergata	Multilevel PLS; KNN
Scarlata 2018 [44]	n = 50 COPD • standard inhalation therapy (12 weeks)		Therapeutic effect	Baseline vs after 12 w Significant decline in VOCs				BIONOTE	PLS-DA
	n = 50 COPD		Clustering	3 clusters Differences: BODE index, number of comorbidities, MEF75, KCO, pH/pCO2 arterial blood					Unsupervised K-means clustering
Van Velzen 2019 [47]	n = 16 AECOPD • before, during and after recovery		Disease course	Before vs during Sens 79% Spec 71% CVA 75%	During vs after Sens 79% Spec 71% CVA 75%		Before vs after Sens 57% Spec 64% CVA 61%	Cyranose 320, Tor Vergata, Comon Invent	PCA
Rodríguez-Aguilar 2020 [40]	n = 116 COPD • n = 88 smoking, n = 28 household air pollution associated • n = 64 GOLD I-II, n = 52 GOLD III-IV n = 178 HC		Diagnostic accuracy	COPD vs HC Sens 100% Spec 97.8% AUC 0.989 Acc 97.8% (CDA), 100% (SVM)	Smoking vs air pollution associated Not significant		GOLD I–II vs GOLD III–IV Not significant	Cyranose 320	PCA; CDA; SVM
***Cystic fibrosis (CF)***									
Paff 2013 [52]	n = 25 CF n = 25 primary ciliary dyskinesia (PCD) n = 23 HC		Diagnostic accuracy	CF vs HC Sens 84% Spec 65% AUC 0.76	CF vs PCD Sens 84% Spec 60% AUC 0.77		Exacerbation CF Sens 89% Spec 56% AUC 0.76	Cyranose 320	PCA
Joensen 2014 [53]	n = 64 CF • n = 14 pseudomonas infection n = 21 PCD n = 21 HC		Diagnostic accuracy	CF vs HC Sens 50% Spec 95% AUC 0.75	CF vs PCD Not significant		Pseudomonas vs. non-infected CF Sens 71.4% Spec 63.3% AUC 0.69 (0.52–0.86)	Cyranose 320	PCA
De Heer 2016 [54]	n = 9 CF colonised *A. fumigatus* n = 18 CF not colonised		Diagnostic accuracy	Sens 78% Spec 94% AUC 0.80–0.89 CVA 88.9%				Cyranose 320	PCA; CDA
Bannier 2019 [23]	n = 13 CF (age > 6 years) n = 22 HC		Diagnostic accuracy	Sens 85% Spec 77% AUC 0.87				Aeonose	ANN
***Interstitial lung disease (ILD)***									
Dragonieri 2013 [58]	n = 31 sarcoidosis • n = 11 untreated • n = 20 treated n = 25 HC		Diagnostic accuracy	Untreated vs HC AUC 0.825 CVA 83.3%	Untreated vs treated CVA 74.2%		Treated vs HC Not significant	Cyranose 320	PCA; CDA
Yang 2018 [59]	*Training:* 80% of n = 34 pneumo-coniosis n = 64 HC	*Validation*: 20% of n = 34 pneumo-coniosis n = 64 HC	Diagnostic accuracy	*Training:* Sens 64.3–67.9% Spec 88.0–92.0% AUC 0.89–0.91 Acc 80.8–82.1%	*Validation*: Sens 33.3–66.7% Spec 71.4–78.6% AUC 0.61–0.86 Acc 65.0–70.0%			Cyranose 320	LDA; SVM
Krauss 2019 [60]	n = 174 ILD • n = 51 IPF • n = 25 CTD-ILD n = 33 HC n = 23 COPD		Diagnostic accuracy	IPF vs HC Sens 88% Spec 85% AUC 0.95	CTD-ILD vs HC Sens 84% Spec 85% AUC 0.90		IPF vs CTD-ILD Sens 86% Spec 64% AUC 0.84	Aeonose	ANN
Dragonieri 2020 [61]	n = 32 IPF n = 36 HC n = 33 COPD		Diagnostic accuracy	IPF vs HC AUC 1.00 (1.00–1.00) CVA 98.5%	IPF vs COPD AUC 0.85 (0.75–0.95) CVA 80.0%		IPF vs COPD + HC AUC 0.84 CVA 96.1%	Cyranose 320	PCA; CDA; LDA
Moor 2020 [57]	*Training:* n = 215 ILD • n = 57 IPF • n = 158 non-IPF n = 32 HC	*Validation*: n = 107 ILD • n = 28 IPF • n = 79 non-IPF n = 15 HC	Diagnostic accuracy	*Training* + *validation*: ILD vs HC Sens 100% Spec 100% AUC 1.00 Acc 100%	*Training:* IPF vs non-IPF ILD Sens 92% Spec 88% AUC 0.91 (0.85–0.96) Acc 91%		*Validation*: IPF vs non-IPF ILD Sens 95% Spec 79% AUC 0.87 (0.77–0.96) Acc 91%	SpiroNose	PLS-DA
***Lung cancer (LC)***									
Machado 2005 [75]	*Training:* n = 14 LC n = 20 HC n = 27 other lung disease	*Validation*: n = 14 LC n = 30 HC n = 32 other lung disease	Diagnostic accuracy	*Training:* LC vs HC + other CVA 71.6% (CDA)	*Validation*: LC vs HC + other Sens 71.4% Spec 91.9% Acc 85% (SVM)			Cyranose 320	SVM PCA CDA
Hubers 2014 [71]	*Training:* n = 20 LC n = 31 HC	*Validation*: n = 18 LC n = 8 HC	Diagnostic accuracy	*Training:* Sens 80% Spec 48%	*Validation*: Sens 94% Spec 13%			Cyranose 320	PCA
Schmekel, 2014 [88]	n = 22 LC • n = 10 survival > 1 year • n = 12 survival < 1 year n = 10 HC		Disease course	< 1 y vs HC R = 0.95–0.98	< 1 y vs > 1 y R = 0.86–0.97		Prediction model survival days R = 0.99	Applied Sensor AB model 2010	PCA; PLS; ANN
McWilliams 2015 [68]	n = 25 LC n = 166 smoking HC		Diagnostic accuracy	Sens 84–96% Spec 63.3–81.3% AUC 0.84				Cyranose 320	Classification and regression tree; DFA
Gasparri 2016 [76]	*Training:* n = 51 LC n = 54 HC	*Validation*: n = 21 LC n = 20 HC	Diagnostic accuracy	*Training* + *validation*: Sens 81% Spec 91% AUC 0.874	*Training:* Sens 90% Spec 100%		*Validation*: Sens 81% Spec 100%	Prototype (8 QMB sensors)	PLS-DA
Rocco 2016 [16]	n = 100 (former) smokers • n = 23 LC		Diagnostic accuracy	Detection LC Sens 86% Spec 95% AUC 0.87				BIONOTE	PLS-Toolbox; PLS-DA
Van Hooren 2016 [81]	n = 32 LC n = 52 head-neck SCC		Diagnostic accuracy	Sens 84–96% Spec 85–88% AUC 0.88–0.98 Acc 85–93%				Aeonose	ANN
Shlomi 2017 [67]	n = 30 benign nodule n = 89 LC • n = 16 early stage LC • n = 53 EGFR tested (n = 19 mutation)		Diagnostic accuracy	Early stage LC vs benign Sens 75% Spec 93.3% Acc 87.0	EGFR mutation vs wild type Sens 79.0% Spec 85.3% Acc 83.0%			Prototype (40 nanomaterial-sensors)	DFA
Tirzite 2017 [83]	n = 165 LC n = 79 HC n = 91 other lung disease		Diagnostic accuracy	LC vs HC + other Sens 87.3–88.9% Spec 66.7–71.2% CVV 72.8%	LC vs HC Sens 97.8–98.8% Spec 68.8–81.0% CVV 69.7%		LC stages Not significant	Cyranose 320	SVM
Huang 2018 [70]	*Training:* 80% of n = 56 LC n = 188 HC	*Validation*: 20% of n = 56 LC n = 188 HC *External:* n = 12 LC n = 29 HC	Diagnostic accuracy	*Validation*: LC vs HC Sens 100, 92.3% Spec 88.6, 92.9% AUC 0.96, 0.95 Acc 90.2, 92.7%	*External validation*: LC vs HC Sens 75, 83.3% Spec 96.6, 86.2% AUC 0.91, 0.90 Acc 85.4, 85.4%			Cyranose 320	LDA; SVM
Van de Goor 2018 [73]	*Training*: n = 52 LC n = 93 HC	*Validation*: n = 8 LC n = 14 HC	Diagnostic accuracy	*Training*: Sens 83% Spec 84% AUC 0.84 Acc 83%	*Validation*: Sens 88% Spec 86% Acc 86%			Aeonose	ANN
Tirzite 2019 [77]	n = 119 LC smoker n = 133 LC non-smoker n = 223 HC + other lung disease • n = 91 smoking		Diagnostic accuracy	LC non-smoker vs HC + other Sens 96.2% Spec 90.6%	LC smoker vs HC + other Sens 95.8% Spec 92.3%			Cyranose 320	LRA
Kononov 2020 [78]	n = 65 LC n = 53 HC		Diagnostic accuracy	Sens 85.0–95.0% Spec 81.2–100% CVA 88.9–97.2% AUC 0.95–0.98				Prototype (6 MOS)	PCA; Logistic regression; KNN; Random forest; LDA; SVM
Krauss 2020 [79]	n = 91 LC active disease • n = 51 incident LC n = 29 LC complete response n = 33 HC n = 23 COPD		Diagnostic accuracy	LC active vs HC Sens 84% Spec 97% AUC 0.92	Incident LC vs HC Sens 88% Spec 79% AUC 89%			Aeonose	ANN
*Lung cancer—(non-)small cell lung cancer ((N)SCLC)*									
Dragonieri 2009 [69]	n = 10 NSCLC n = 10 COPD n = 10 HC		Diagnostic accuracy	NSCLC vs HC CVV 90%	NSCLC vs COPD CVV 85%			Cyranose 320	PCA; CDA
Kort 2018 [72]	n = 144 NSCLC n = 18 SCLC n = 85 HC n = 61 suspected, LC excluded		Diagnostic accuracy	NSCLC vs HC Sens 92.2% Spec 51.2% AUC 0.85	NSCLC vs HC + LC excluded Sens 94.4% Spec 32.9% AUC 0.76		SCLC vs HC Sens 90.5% Spec 51.2% AUC 0.86	Aeonose	ANN
De Vries 2019 [87]	*Training*: n = 92 NSCLC • n = 42 response • n = 50 no response	*Validation*: n = 51 NSCLC • n = 23 response • n = 28 no response	Therapeutic effect (anti-PD-1 therapy)	*Training*: CVV 82% AUC 0.89 (0.82–0.96)	*Validation*: AUC 0.85 (0.7–0.96) Sens 43% Spec 100%			SpiroNose	LDA
Mohamed 2019 [80]	n = 50 NSCLC n = 50 HC		Diagnostic accuracy	Sens 92.9% Spec 90% Acc 97.7%				PEN3	PCA; ANN
Kort 2020 [74]	n = 138 NSCLC n = 143 controls • n = 59 suspected, LC excluded • n = 84 HC		Diagnostic accuracy	NSCLC vs controls (eNose data only) Sens 94.2% Spec 44.1% AUC 0.75	NSCLC vs controls (multivariate) Sens 94.2–95.7% Spec 49.0–59.7% AUC 0.84–0.86			Aeonose	ANN; Multivariate logistic regression
Fielding 2020 [82]	n = 20 bronchial SCC • n = 10 in situ • n = 10 advanced stage n = 22 laryngeal SCC • n = 12 in situ • n = 10 advanced stage n = 13 HC		Diagnostic accuracy	BSCC in situ vs HC Sens 77% Spec 80% Misclassification rate 28%	BSCC vs LSCC adv Sens 100% Spec 80% Misclassification rate 10%			Cyranose 320	Bootstrap forest
*Lung cancer—Malignant Pleural Mesothelioma (MPM)*									
Chapman 2012 [86]	*Training*: n = 10 MPM n = 10 HC	*Validation*: n = 10 MPM n = 32 HC n = 18 benign ARD	Diagnostic accuracy	MPM vs HC *Training:* CVA 95% *Validation*: Sens 90% Spec 91%	MPM vs ARD *Validation*: Sens 90% Spec 83.3%		MPM vs ARD vs HC *Validation*: Sens 90% Spec 88%	Cyranose 320	PCA
Dragonieri 2012 [85]	n = 13 MPM • internal validation with *training set*: n = 8, *validation set*: n = 5 n = 13 HC n = 13 AEx		Diagnostic accuracy	MPM vs HC Sens 92.3% Spec 69.2% AUC 0.893 CVA 84.6% *Validation*: AUC 0.83 CVA 85.0%	MPM vs AEx Sens 92.3% Spec 85.7% AUC 0.917 CVA 80.8% *Validation*: AUC 0.88 CVA 85.9%		MPM vs AEx vs HC AUC 0.885 CVA 79.5%	Cyranose 320	PCA; CDA
Lamote 2017 [84]	n = 11 MPM n = 12 HC n = 15 AEx n = 12 benign ARD		Diagnostic accuracy	MPM vs HC Sens 66.7% (37.7–88.4) Spec 63.6% (33.7–87.2) AUC 0.667 (0.434–0.900) Acc 65.2% (44.5–82.3)	MPM vs benign ARD Sens 75.0% (45.9–93.2) Spec 64% (33.7–87.2) AUC 0.758 (0.548–0.967) Acc 48.9–85.6% (48.9–85.6)		MPM vs benign ARD + AEx Sens 81.5% (63.7–92.9) Spec 54.5% (26.0–81.0) AUC 0.747 (0.582–0.913) Acc 73.7% (58.1–85.8)	Cyranose 320	PCA
***Pulmonary infections***									
De Heer 2016 [100]	n = 168 bottles with strain • n = 135 bacteria + yeast • n = 30 medium only • n = 62 mould (*A. fumigatus* and *R. oryzae*)		Diagnostic accuracy (in vitro)	Mould vs other Sens 91.9% Spec 95.2% AUC 0.970 (0.949–0.991) Acc 92.9%				Cyranose 320	PCA; CDA
Suarez-Cuartin 2018 [101]	n = 73 bronchiectasis • n = 41 colonised (n = 27 pseudomonas) • n = 32 non-colonised		Diagnostic accuracy	Colonised vs non-colonised AUC 0.75 CVA 72.1%	Pseudomonas vs other PPM AUC 0.96 CVA 89.2%		Pseudomonas vs non-colonised AUC 0.82 CVA 72.7%	Cyranose 320	PCA
*Pulmonary infections—Ventilator-associated pneumonia (VAP)*									
Hanson 2005 [104]	n = 19 VAP (clinical pneumonia score, CPIS ≥ 6) n = 19 controls (CPIS < 6)		Diagnostic accuracy	Correlation CPIS -breathprint R^2^ = 0.81				Cyranose 320	PLS
Hockstein 2005 [105]	n = 15 VAP (pneumonia score ≥ 7) n = 29 HC (ventilated)		Diagnostic accuracy	Acc 66–70%				Cyranose 320	KNN
Humphreys 2011 [99]	n = 44 VAP suspected • 98 BAL samples • Groups: gram-positive, gram-negative, fungi, no growth n = 6 HC (ventilated)		Diagnostic accuracy (in vitro)	Differentiation groups (LDA) Sens 74–95% Spec 77–100% Acc 83%	Differentiation groups (cross-validation) Sens 56–84% Spec 81–97% Acc 70%			Prototype (24 MOS)	PCA; LDA
Schnabel 2015 [106]	n = 72 VAP suspected • n = 33 BAL + • n = 39 BAL− n = 53 HC (ventilated)		Diagnostic accuracy	BAL + VAP vs HC Sens 88% Spec 66% AUC 0.82 (0.73–0.91)	BAL + vs BAL− VAP Sens 76% Spec 56% AUC 0.69 (0.57–0.81)			DiagNose	Random Forest; PCA
Chen 2020 [15]	*Training:* 80% of n = 33 VAP n = 26 HC (ventilated)	*Validation*: 20% of n = 33 VAP n = 26 HC (ventilated)	Diagnostic accuracy	*Training:* AUC 0.823 (0.70–0.94)	*Validation*: Sens 79% (± 8) Spec 83% (± 0) AUC 0.833 (0.70–0.94) Acc 0.81 (± 0.04)			Cyranose 320	KNN; Naive Bayes; decision tree; neural network; SVM; random forest
*Pulmonary infections—Tuberculosis (TB)*									
Fend 2006 [109]	n = 188 TB n = 142 TB excluded		Diagnostic accuracy (in vitro)	Sens 89% (80–97) Spec 88% (85–97)				Bloodhound BH-114	PSA; DFA; ANN
Bruins 2013 [107]	*Training:* n = 15 TB n = 15 HC	*Validation*: n = 34 TB n = 114 TB excluded n = 46 HC	Diagnostic accuracy	*Training:* Sens 95.9% (92.9–97.7) Spec 98.5% (96.2–99.4)	*Validation*: TB vs HC Sens 93.5% (91.1–95.4) Spec 85.3% (82.7–87.5)		*Validation*: TB vs TB excl Sens 76.5% (57.98–88.5) Spec 74.8% (64.5–82.9)	DiagNose	ANN
Coronel Teixeira 2017 [108]	*Training:* n = 23 TB n = 46 HC	*Validation*: n = 47 TB n = 63 HC + asthma + COPD	Diagnostic accuracy	*Training:* Sens 91% Spec 93%	*Validation*: Sens 88% Spec 92%			Aeonose	Tucker 3–like algorithm; ANN
Mohamed 2017 [110]	n = 67 TB n = 56 HC		Diagnostic accuracy	Sens 98.5% (92.1–100) Spec 100% (93.5–100) Accuracy 99.2%				PEN3	PCA; ANN
Saktiawati 2019 [111]	*Training:* n = 85 TB n = 97 HC + TB excluded	*Validation*: n = 128 TB n = 159 TB excluded	Diagnostic accuracy	*Training:* Sens 85% (75–92) Spec 55% (44–65) AUC 0.82 (0.72–0.88)	*Validation*: Sens 78% (70–85) Spec 42% (34–50) AUC 0.72 (0.66–0.78)			Aeonose	ANN
Zetola 2017 [112]	n = 51 TB n = 20 HC		Diagnostic accuracy	Sens 94.1% (83.8–98.8) Spec 90.0% (68.3–98.8)				Prototype (QMB sensors)	PCA; KNN
*Pulmonary infections—Aspergillosis*									
De Heer 2013 [102]	n = 11 neutropenia • n = 5 probable/proven aspergillosis • n = 6 no aspergillus		Diagnostic accuracy	Sens 100% (48–100) Spec 83.3% (36–100) AUC 0.933 CVA 90.9% (59–100)				Cyranose 320	PCA; CDA
De Heer 2016 [54]	n = 9 CF colonised *A. fumigatus* n = 18 CF not colonised		Diagnostic accuracy	Sens 78% Spec 94% AUC 0.80–0.89 CVA 88.9%				Cyranose 320	PCA; CDA
*Pulmonary infections—Corona Virus Disease (COVID-19)*									
Wintjens 2020 [114]	n = 219 screened • n = 57 COVID-19 positive		Diagnostic accuracy	Sens 86% (74–93) Spec 54% (46–62) AUC 0.74 CVA 62%				Aeonose	ANN
***Obstructive sleep apnoea (OSA)***									
Greulich 2013 [89]	n = 40 OSA n = 20 HC		Diagnostic accuracy	OSA vs HC Sens 93% Spec 70% AUC 0.85				Cyranose 320	PCA
	N = 40 OSA • 3 months CPAP ventilation		Therapeutic effect	Before vs after CPAP Sens 80% Spec 65% AUC 0.82					
Incalzi 2014 [95]	n = 50 OSA • 1 night CPAP ventilation		Therapeutic effect	Change in breathprint (visually different, no statistical analysis)				BIONOTE	PCA; PLS-DA
Dragonieri 2015 [90]	n = 19 OSA n = 14 obese n = 20 HC		Diagnostic accuracy	Obese OSA vs HC CVA% 97.4 AUC 1.00	Obese OSA vs obese CVA% 67.6 AUC 0.77		Obese vs HC CVA% 94.1 AUC 0.94	Cyranose 320	PCA; CDA; KNN
Kunos 2015 [96]	n = 17 OSA n = 9 non-OSA sleep disorder n = 10 HC • 7AM and 7PM sample n = 26 HC –7AM sample		Diagnostic accuracy	OSA 7AM vs 7PM Significantly different	Non-OSA or HC 7AM vs 7PM Not significantly different		(Non-)OSA 7AM vs HC 7AM Significantly different Acc 77–81%	Cyranose 320	PCA
Dragonieri 2016 [92]	*Training:* n = 13 OSA n = 15 COPD n = 13 overlap	*Validation*: n = 6 OSA n = 6 COPD n = 6 overlap	Diagnostic accuracy	*Training:* OSA vs overlap CVA 96.2% AUC 0.98	*Validation*: OSA vs overlap CVA 91.7% AUC 1.00		*Validation*: OSA vs COPD CVA 75% AUC 0.83	Cyranose 320	PCA; CDA
Scarlata 2017 [91]	n = 40 OSA • n = 20 hypoxic n = 20 obese n = 20 COPD n = 56 HC		Diagnostic accuracy	OSA vs HC Acc 98–100%	Non-hypoxic vs hypoxic OSA Acc 60–80%		HC vs COPD Acc 100%	BIONOTE	PLS-DA
***Other—Acute respiratory distress syndrome (ARDS)***									
Bos 2014 [115]	*Training:* n = 40 ARDS n = 66 HC	*Validation*: n = 18 ARDS n = 26 HC	Diagnostic accuracy	*Training:* Sens 95% Spec 42% AUC 0.72	*Validation*: Sens 89% Spec 50% AUC 0.71			Cyranose 320	Sparse-partial least square logistic regression
***Other—Lung transplantation (LTx)***									
Kovacs 2013 [117]	n = 16 LTx recipients n = 33 HC		Diagnostic accuracy	LTx recipients vs HC Sens 63% Spec 75% AUC 0.825				Cyranose 320	PCA; Linear regression
			Therapeutic effect	Correlation breathprint—tacrolimus levels R = -0.63				Cyranose 320	PCA; Linear regression
***Other—Pulmonary embolism (PE)***									
Fens 2010 [116]	n = 20 PE • n = 7 comorbidity n = 20 PE excluded • n = 13 comorbidity		Diagnostic accuracy	Comorbidity: PE vs excluded Acc 65% AUC 0.55	No comorbidity: PE vs excluded Acc 85% AUC 0.81		No comorbidity: PE vs excluded (breathprint + Wells) AUC 0.90	Cyranose 320	PCA

An overview of eNose technology studies in lung diseases. Studies are divided per diagnosis and displayed in chronological order. Study results shown in sensitivity/specificity, AUC and CVA (if available). In case of a training and validation set, participant numbers and results of both set are shown. All presented results are statistical significant (p < 0.05) unless stated otherwise

*AATd * alpha-1-antitrypsin deficiency, *acc* accuracy, *AUC*  area under the curve, *AAR*  extrinsic asthma with allergic rhinitis, *AEx*  asbestos exposure, *ANN*  artificial neural network, *ARD*  benign asbestos related disease, *BMI*  body mass index, *CDA*  canonical discriminant analysis, *CVA/CV*V  cross-validated accuracy/value, *d*  days, *DFA*  discriminate function analysis, *EBC*  exhaled breath condensate, *AECOPD*  acute COPD exacerbation, *EGFR*  epidermal growth factor receptor, *eos*  eosinophils, *FeNO*  exhaled nitric oxide test, *FVC*  forced vital capacity, *GOLD*  global initiative for chronic obstructive lung disease, *HC*  healthy control (not suspected for studied disease, not diagnosed with other pulmonary disease), *ICS*  inhaled corticosteroids, *IPF*  idiopathic pulmonary fibrosis, *KNN*  k-nearest neighbours, *LDA*  linear discriminant analysis, *MOS*  metal oxide sensor, *n6MWD*  normalised six minute walking distance, *OCS*  oral corticosteroids, *PAM*  partitioning around medoids, *PCA*  principal component analysis, *PEB*  pure exhaled breath, *PLS-DA*  partial least squares discriminant analysis, *PPM*  potentially pathogenic microorganism, *QMB*  quartz microbalance, *QoL*  quality of life, *ROC* receiver operator characteristics, *SCC*  squamous cell carcinoma (*B*  bronchial, *L*  laryngeal), *sens*  sensitivity, *SLR*  Sensor Logic Relations, *spec*  specificity, *SVM*  support vector machines, *TLC* total lung capacity

### Asthma

Asthma is a chronic lung disease characterised by reversible airflow obstruction with airway inflammation and hyperresponsiveness. Common symptoms, such as cough, chest tightness, shortness of breath and wheezing, are variable in severity and often non-specific [17]. Various studies, both in children and adults, showed that eNose technology can differentiate asthma patients from healthy controls with a good accuracy [18–25]. Two studies also demonstrated that breathprints of asthma patients were significantly different than breathprints of chronic obstructive pulmonary disease (COPD) patients [19, 26]. Interestingly, two studies reported better performance of eNose technology than conventional investigations (spirometry or an exhaled nitric oxide (FeNO) test) for detecting asthma. These studies were performed in patients with an established asthma diagnosis [21, 22]. Diagnostic performance further increased when eNose technology was combined with a FeNO test (accuracy 95.7%) [21]. Moreover, even after loss of control and reaching stable disease with oral corticosteroids (OCS) treatment eNose technology could differentiate asthma from healthy controls, while the diagnostic value of FeNO decreased. In the same study, breathprint significantly predicted response to subsequent OCS treatment, while sputum eosinophils, FeNO values and, hyperresponsiveness did not [22].

The existence of multiple asthma pheno- and endotypes with different underlying pathophysiological mechanisms is increasingly acknowledged [27]. In recent years, many eNose studies have attempted to identify different clusters of asthma patients, using both supervised and unsupervised methods [28–31]. For example, supervised clustering for eosinophilic, neutrophilic and paucigranulocytic phenotypes revealed significant differences in breathprints between groups [30]. One study identified three clusters using unsupervised breathprint analysis in a group of severe asthmatic patients, corresponding with different inflammatory profiles. During follow-up, 30 of 51 patients migrated to another cluster; migration was associated with changes in sputum eosinophil count [31]. Two other longitudinal studies showed changes in breathprint when asthma control was lost after withdrawal of corticosteroids in previously stable asthma patients, and also after recovery [22, 32]. A pilot study, in which bronchoconstriction was induced in stable asthma patients, found that changes in airway calibre did not alter breathprints. Moreover, breathprints remained stable during the day in individual patients [20]. This implies that inflammatory processes and not (acute) airway obstruction influence breathprints. Overall, these findings suggest that eNose technology is a promising tool for phenotyping and monitoring asthmatics. Longer follow-up studies are required to examine whether cluster-migration or change in breathprint are also related to actual clinical course.

A currently ongoing study is evaluating whether eNose technology can be used to predict response to monoclonal antibody therapy (NCT03988790).

#### Paediatric asthma

In general, the diagnosis of asthma in children is challenging. Lung function tests are often difficult to perform and do not always provide a diagnosis. Interestingly, a study in 45 children demonstrated that eNose measurements were fairly well repeatable, both in healthy and asthmatic participants [33].

Moreover, two studies showed that eNose technology distinguishes children with asthma from healthy controls [23, 25, 34].﻿ An eNose seemed to be more accurate for diagnosing asthma than spirometry with bronchodilation only [34]. Also, uncontrolled asthma could be differentiated from controlled asthma and healthy controls [25﻿]. Furthermore, eNose technology accurately distinguished children with persistent asthma from healthy controls, but not the ones with intermittent asthma [34]. This was possibly due to more airway inflammation reflected in the breathprints of persistent asthmatics. Hence, eNose technology could potentially facilitate easier and earlier diagnosis of asthma in children, and guide therapy in clinical practice. However, large validation studies focusing on diagnosing asthma in children are currently lacking.

### COPD

Although COPD is one of the major causes of death worldwide, epidemiological studies indicate that it remains largely underdiagnosed [35]. COPD is a complex, heterogeneous disease with several phenotypes, which can overlap with asthma and pulmonary infections, among others. Furthermore, the diagnosis is delayed in patients whose symptoms are attributed to (undiagnosed) heart failure [36]. Hence, there is an unmet clinical need for accurate timely diagnosis. Also better disease course prediction and therapy guidance is warranted.

Several studies have evaluated the ability of eNose technology to diagnose COPD. Exhaled breath analysis discriminated between COPD and (smoking) healthy controls with an accuracy of 66–100% [19, 37–41]. Even though these are promising results, most studies were relatively small and lacked a validation cohort. Several studies aimed to distinguish subgroups within COPD by performing unsupervised analyses on breathprint data [42–44]. De Vries et al. performed unsupervised cluster analysis in a combined group of asthma and COPD patients [43]. Interestingly, they identified and validated five clusters which mainly differed based on clinical and inflammatory characteristics (eosinophil and neutrophil count) rather than diagnosis. Two other studies identified 3–4 unsupervised clusters based on breathprint data. The clusters differed regarding several clinical and demographic features [42, 44]. However, in both studies, clusters were determined by different clinical parameters, showing the need for further (validation) studies. A recent study indicated that breathprints of patients with COPD associated with air pollution did not differ from smoking-associated COPD [40]. Also, no differences in breathprint between Global Initiative for Chronic Obstructive Lung Disease (GOLD) stage I-II versus GOLD stage III-IV were detected in another study [40]. The breathprint of patients with smoking-related COPD and patients with alpha-1-antitripsin, however, could be distinguished with an accuracy of 82% in a small single-centre study [37].

eNose technology can theoretically be useful in early detection of inflammation and acute exacerbation of COPD (AECOPD), as inflammatory processes influence breathprints. This hypothesis was confirmed in a cross-sectional study evaluating the association of breathprints with different inflammation markers in sputum; eNose breathprints highly correlated with inflammatory activity [45]. In patients with an AECOPD, presence of viral and bacterial infection was accurately detected by an eNose [46]. In another group of AECOPD patients, patients with colonisation of potentially pathogenic microorganisms had a significantly different breathprint than AECOPD patients that were not colonised. Besides, AECOPD patients’ breathprints differed from stable COPD patients without microorganism colonisation [39]. Stable COPD patients with bacterial colonisation were also significantly different from those without (area under the curve (AUC) 0.922) [41]. Two prospective longitudinal studies indicated that the breathprint before, during and after recovery of an AECOPD differed [39, 47]. Confirming these results in larger cohort studies might lead the way to use breathprints for earlier detection and (targeted) treatment of infections and AECOPDs. This is interesting as treatment may improve outcomes and prevent hospitalizations [48].

Regarding prognostic value of eNose technology, one study demonstrated that eNose data correlated better to change in 6-min walking distance over one year, than the current GOLD classification [49]. A few studies evaluated the effect of initiation and withdrawal of inhalation medication on breathprints. Two studies found significant changes in breathprint after start of inhalation therapy [44, 50]. A designed multidimensional model, combining eNose technology with spirometry, gave a better indication of treatment response (AUC 0.857) than spirometry only (AUC 0.561) [50]. This small pilot study shows the potential of integrating eNose technology in standard practice. However, it remains to be elucidated whether eNose technology can serve as a marker for therapy compliance of inhaled medication.

### Cystic fibrosis

Cystic fibrosis (CF) is associated with bronchiectasis, recurrent infectious exacerbations, and progressive deterioration of lung function due to exacerbations [51].

A few studies using different eNoses showed that patients with CF could accurately be distinguished from healthy controls and asthma patients based on their breathprint [23, 52, 53]. Two studies showed conflicted results regarding differentiation of CF from primary ciliary dyskinesia (PCD) patients, a bronchiectatic lung disease that mimics symptoms of CF [53]. While Paff et al. showed that CF and PCD could be adequately discriminated, Joensen et al. found no significant differences [52, 53]. This was possibly due to methodological differences, such as different breath collection methods and a more heterogeneous patient population in the latter study. Furthermore, eNose technology adequately discriminated between patients with and without exacerbations, with and without chronic Pseudomonas aeruginosa colonisation, and patients with and without Aspergillus fumigatus colonisation [52–54]. It would be of great interest to investigate whether early stage respiratory infections and exacerbations can also be detected and eventually be predicted by eNose technology. This will possibly increase the chance of successful eradication and slowing down pulmonary function decline.

### Interstitial lung disease

Interstitial lung disease (ILD) is a heterogeneous group of relatively uncommon diseases causing fibrotic and/or inflammatory changes in interstitial lung tissue. Disease course and treatment strategies widely vary for different ILDs, and even within individual ILDs disease course often varies. Diagnosis is based on integration of clinical data with imaging and if needed pathology data. Diagnosis is often complex and diagnostic delays are common [55, 56]. eNose technology has the potential to replace invasive procedures, and aid the diagnostic process to facilitate timely and accurate diagnosis.

A large single centre cohort, including various ILDs, found that breathprints of ILD patients could be distinguished from healthy controls with 100% accuracy. Results were confirmed in a validation cohort [57]. A few other studies compared individual ILDs with healthy controls and COPD patients [58–61]. Breathprints of patients with idiopathic pulmonary fibrosis (IPF), ILD associated with connective tissue disease and pneumoconiosis were significantly different from healthy controls [59–61]. In sarcoidosis patients, the breathprint of patients with untreated sarcoidosis differed from healthy controls, implying that eNose technology may be used for initial diagnosis. This study found that breathprints of treated sarcoidosis patients were not significantly different from healthy controls, but the number of participants was small [58]. Comparing different ILDs, eNose technology distinguished IPF from non-IPF ILD patients with an accuracy of 91% in both training and validation cohort. Exploratory analyses indicated that individual ILDs can also be discriminated adequately [57]. However, groups were relatively small and, thus, results should be validated and confirmed in larger cohorts. A currently ongoing large multicentre study is investigating the potential of eNose technology to identify individual diseases, predict disease course, and response to treatment in fibrotic ILDs (NCT04680832).

### Lung cancer

Worldwide, lung cancer is the leading cause of cancer deaths and has the highest incidence of all cancer types. More than 80% of patients suffering from lung cancer are former or current tobacco smokers [62]. Early diagnosis is clearly associated with better outcomes, and lung cancer screening has shown to reduce mortality [63, 64]. Nevertheless, early diagnosis remains challenging, since initial clinical presentation often overlaps with COPD or other smoking-related diseases, and symptoms often only appear in late stages [65]. Low-dose CT scan is currently the best available tool for screening. However, this type of screening is only cost-effective in a selected group of former and current smokers [66]. Also, differentiation of benign from malignant nodules is not possible with CT scan results; therefore, detected nodules warrant further invasive investigations. eNose could possibly serve as non-invasive and less costly screening tool to identify malign pulmonary neoplasms. Two studies used eNose technology in high-risk patients enrolled for lung cancer screening. Both studies found a higher specificity for detecting lung cancer with eNose compared to low-dose CT scan; thus, the use of eNose technology as screening tool can potentially reduce the false-positive rate and prevent unnecessary (invasive) testing [16, 67]. It is important to note that not all lesions classified as benign were histologically proven in these studies.

Whether an eNose can differentiate lung cancer patients from healthy controls, patients with benign lung nodules or (former) smokers, has been investigated in different cohorts. All studies in (non-) small cell lung cancer ((N)SCLC) showed significant results, albeit with a wide range in reported sensitivity (71–99%) and specificity (13–100%) [68–80]. Smoking status of participants did not seem to influence accuracy of an eNose for detecting cancer [77]. One small study showed that patients with and without an EGFR (epidermal growth factor receptor) mutation had distinct breathprints [67]. It has not been evaluated whether eNoses can ﻿recognise specific types of lung cancer in a cohort with different subtypes. Recognition of subtypes seems plausible, as differentiation of lung cancer from head-neck cancer was possible with eNose technology [81, 82]. eNose technology did not discriminate between different stages of lung cancer [83]. One recent study in NSCLC combined eNose data with relevant clinical parameters (such as age, number of pack years, and presence of COPD), and showed a higher accuracy for lung cancer detection than using eNose data only. These results highlight the potential of eNose technology as additional diagnostic procedure [74]. Some small studies indicated that eNose technology was also able to differentiate patients suffering from malignant pleural mesothelioma (MPM) and healthy controls. Differentiation of MPM from benign asbestosis disease and asymptomatic asbestos exposure had a high sensitivity too [84–86].

Prediction of response to therapy is investigated for anti-programmed death (PD)-1 receptor therapy in NSCLC patients. Breathprints were collected before start of pembrolizumab or nivolumab therapy. Exhaled breath data could predict which patients would respond to therapy with an AUC of 0.89, confirmed in a validation cohort. By setting a cut-off value to obtain 100% specificity, the investigators were able to detect 24% of non-responders to anti-PD-1 therapy. In this regard, eNose seems to be more accurate than the currently used biomarker PD-L1 [87]. Another study is currently registered for recruiting until July 2021 and will evaluate the effect of immunotherapy on breathprints of exhaled breath and sweat in lung cancer patients (NCT03988192).

Schmekel et al. investigated the ability of eNose to predict prognosis in patients with end stage lung cancer. They collected breathprints before start and several times after start of palliative chemotherapy and applied different prediction models. Patients with less than one year survival and more than one year survival could be separated based on breathprint [88]. The authors suggest to use this eNose-based prediction for choosing a certain treatment strategy, but this needs confirmation in studies first.

### Obstructive sleep apnoea

At the moment, the gold standard for diagnosing obstructive sleep apnoea (OSA) is (poly)somnography which is a costly and time-consuming test. eNose technology has been investigated as an alternative modality to diagnose this condition and assess treatment effect.

It was shown that breathprints from OSA patients and healthy controls can be distinguished reliably [89–91]. However, it remains questionable whether breathprints distinguishes true OSA, or if the breathprint is just a reflection of a metabolic syndrome or underlying inflammation caused by obesity. In one of the studies this question was more apparent as groups were not matched for body mass index [89]. Dragonieri et al. found that eNose technology did discriminate obese patients with and without OSA, with moderate accuracy [90]. Nevertheless, another study could not confirm those results [91].

Other researchers investigated OSA, OSA-COPD overlap syndrome and COPD. OSA could be distinguished from the overlap syndrome, but eNose technology could not discriminate well between the overlap syndrome and COPD. Also here it is not clear whether true OSA can be detected or other factors, such as COPD, are picked up [91, 92]. Whether included patients also suffer from heart failure is not clearly displayed in these studies, although it is known that many heart failure patients suffer from OSA and that heart failure might influence breathprint [93, 94].

The effects of continuous positive airway pressure (CPAP) treatment in patients with OSA has also been studied. The breathprint of OSA patients changed significantly already after one night of CPAP treatment [95]. Significant difference in breathprint was also found before and after three months of CPAP treatment [89]. It remains to be elucidated what this change in breathprint indicates. Possibly, the alteration in breathprint could serve as a marker for metabolic success, therapeutic benefit or treatment adherence. Furthermore, it must be noted that the breathprints of patients with OSA differed between morning and evening [96]. Hence, diurnal variance must be taken into account when using an eNose for patients with OSA.

### Pulmonary infections

Pathogenic micro-organisms, such as viruses, bacteria or fungi, can cause severe pulmonary infections. Identification of specific micro-organisms with sputum cultures can take up to several days, and is only possible if a specimen with sufficient quality is obtained. Specificity and sensitivity also depend on the causative micro-organism, experience of laboratory observer, and prior treatment [97]. Therefore, reported sensitivity of detecting bacteria in sputum culture ranges between 57 and 95%, and specificity between 48 and 87% [98]. Detection of specific micro-organisms using eNose technology can potentially reduce misuse of antibiotics and facilitate timely start of guided therapy.

Until now, two in vitro studies aimed to differentiate micro-organisms by analysing breathprints of their headspace air [99, 100]. Mould species were discriminated from other samples (bacteria, yeasts, and control medium) with a high accuracy (92.9%). Furthermore, different mould species seemed to have different breathprints [100]. Another study performed eNose analyses on bronchoalveolar lavage samples, and demonstrated accurate discrimination between Gram-positive bacteria, Gram-negative bacteria, fungi, and samples without growth of micro-organisms [99]. In vivo*,* breathprints of bronchiectasis patients significantly differed between those colonised with Pseudomonas Aeruginosa and those colonised with other pathogenic micro-organisms or non-colonised [101]. For detection of aspergillus colonisation or invasive aspergillosis in specific patient groups (CF and neutropenic patients), studies revealed a high accuracy of eNose breathprint analysis [54, 102]. These studies did not include a validation cohort or healthy control group.

Ventilator-associated pneumonia (VAP) is a common nosocomial infection in ventilated patients and has an incidence and mortality around 9% [98, 103]. In most eNose studies, bacterial growth in sputum or a clinical pneumonia score was used to define VAP [15, 104–106]. Two studies showed that obtained breathprints highly correlated with a clinical pneumonia score, implying that eNose technology might be used to predict the probability of a VAP [104, 105]. Two case–control studies in patients with VAP and ventilated patients without pneumonia showed conflicting results; Schnabel and colleagues concluded that eNose technology lacked sensitivity and specificity, whereas a recently published study of Chen and colleagues found a good accuracy for detecting VAP [15, 106]. This shows the need for more research on this topic before eNose can be used to determine the need for more (invasive) diagnostics in ill patients, such as performing bronchoscopy.

In pulmonary tuberculosis (TB) patients, detection and screening with eNose technology has been studied in different countries and compared to different control groups [107–112]. As TB is the leading cause of death from an infection caused by a single micro-organism, and as it has a high prevalence in developing countries, establishing a fast non-invasive cheap screening tool is much needed [113]. In one study, eNose technology differentiated TB from non-TB quite accurately, suggesting that it can potentially serve as a screening tool. Detection of TB had a sensitivity of 89% and a specificity of 91% compared to positive cultures. This sensitivity and specificity exceeded Ziehl–Neelsen staining [109]. However, all studies with proven TB and healthy participants in the training cohort, had a lower accuracy when validating the results in a cohort also including suspected TB patients [107, 108, 111]. Thus, more research is necessary before eNose technology can be used as a population-wide screening tool.

Due to the Corona Virus Disease (COVID-19) pandemic, much research effort is being put in the evaluation of eNose technology as a fast and non-invasive tool for the detection of COVID-19 (NCT04475562, NCT04475575, NCT04558372, NCT04379154, NCT04614883, NL8694). To date, one study tested the accuracy of eNose technology for COVID-19 screening prior to surgery in non-symptomatic patients and found a negative predictive value up to 0.96. Reverse transcription-polymerase chain reaction on a pharyngeal swab and antibody testing were used to confirm presence or absence of COVID-19 [114].

### Other

A number of eNose studies have been performed in other lung diseases. In acute respiratory distress syndrome (ARDS), eNose technology could discriminate between mechanically ventilated patients with and without ARDS, with moderate accuracy in a training and validation cohort [115].

One small proof-of-principle study has been performed in patients with suspected pulmonary embolism, defined as a high clinical probability according to the Well’s score or elevated D-dimer. Breathprints of non-comorbid patients with and without pulmonary embolism could be distinguished with an accuracy of 85%. However, in patients with comorbidities known to influence VOCs (e.g. cancer, diabetes) the accuracy dropped [116].

Finally, eNose technology could be useful for follow-up and monitoring lung transplant recipients. One study found a significant association between breathprint and plasma tacrolimus levels, suggesting that eNoses might be used for non-invasive therapeutic drug monitoring [117].

A clinical trial in lung transplant recipients is currently conducted (NL9251) looking at discrimination of stable lung transplant recipients, acute cellular rejection, and chronic lung allograft rejection.

## Discussion

In the past decades, multiple eNoses have been developed and tested in numerous clinical studies for a wide spectrum of lung diseases. So far, the vast majority of studies evaluated the ability of eNose technology to distinguish lung diseases from healthy controls, and to discriminate between different diagnoses. A small number of studies have been performed for prognostic or therapeutic purposes, and only a handful of studies have focused on clustering patients by breathprint and identifying phenotypes. Results in lung diseases are overall very promising, but several issues should be addressed before eNoses can be implemented in daily clinical practice.

One of the issues is the use of various eNose devices with different qualifications, types of sensors and breath sample collection methods as summarised in Table 1. It is not possible to point out the best eNose device or select one optimal sensor type, as each setting, disease and research aim can require different features. For example, a portable device might be optimal for an acute care setting, direct sampling without collection bags might be useful in low resource areas and as point-of-care technique, and a device that corrects for ambient air will probably generate more comparable results in multicentre use and settings with unstable or varying environmental conditions.

Given important differences between the various devices, it is difficult to compare data of the different eNose devices. Hence, each eNose needs to be validated for every clinical application. This implies that knowledge about characteristics of eNose devices is essential before initiating eNose research, as the type of device cannot easily be changed during the trajectory of developing a clinical tool. Additionally, the influence of endogenous (e.g. comorbidities, ethnicity, age) and exogenous factors (e.g. smoking, nutrition, drug use, measurement environment) on breathprints needs to be further elucidated.

Furthermore, studies differ significantly with regards to study design (e.g. patient selection, number of participants, and presence of a validation cohort). As illustrated in Fig. 2, the majority of studies so far can be considered as pilot or exploratory studies, and have small numbers of participants. The most important goal of these studies is to test new hypotheses, which can be further assessed and confirmed in larger studies with external validation. However, these validation studies are not often conducted. This lack of validation is a major issue in development of a clinical useful breath biomarker, as breath analysis results are not always interchangeable between research settings due to a combination of the above mentioned factors. To ensure optimal outcomes, comparison and generalisability of eNose studies, the design and analysis methods should ideally be based on specific predefined research aims.

Moreover, most studies do not explain the rationale for choosing a certain machine learning model for analysing eNose data. This prevents insights in and discussion regarding the optimal analysis techniques and algorithms. Machine learning models are complex to execute and interpret, and if not used in the right way are prone for overfitting. To avoid inadequate modelling, data scientists should always be involved in these complex analyses and models should be validated independently to exclude overfitting. To allow for comparison of different modelling techniques, we recommend an extensive world-wide shared database per eNose with FAIR (findable, accessible, interoperable, and reusable) and open source data, including patient characteristics and other pre-test probabilities. This database would ensure optimal training, validation, and application of models.

Finally, a factor that hampers eNose implementation is the need for a strong gold standard to establish a diagnosis or to evaluate therapeutic effect. High quality data input is required for optimal validity when developing a new technique. Some of the diseases mentioned in this review lack a gold standard, and even if a gold standard does exist, there is always a range of uncertainty. There is a potential for unsupervised machine learning models in this regard, as such analyses could help to identify previously unrecognised phenotype clusters. Discovering such new clusters can help to generate hypotheses about the existence of unravelled disease subtypes or overlap between diagnoses, and might eventually guide new diagnostic standards.

In conclusion, eNose technology in the field of lung diseases is promising and at the doorstep of the pulmonologist’s office. To facilitate clinical implementation, we recommend conducting prospective multicentre trials including validation in external cohorts with a study design and analysis method relevant for the research aim, and sharing databases on open source platforms. If supported by sufficient evidence, research can subsequently be extended to clinical implementation studies, and finally, use in daily practice.

We believe that eNose technology has the potential to facilitate personalised medicine in lung diseases through establishing early, accurate diagnosis and monitoring disease course and therapeutic effects.

## Supplementary Information

**Additional file 1.** Sensor technology explained.

**Additional file 2.** Search strategy.

## Data Availability

The data supporting the conclusions of this article are included in this published article.

## Abbreviations

AECOPD: Acute exacerbation of COPD
AUC: Area under the curve
ARDS: Acute respiratory distress syndrome
CF: Cystic fibrosis
COPD: Chronic obstructive pulmonary disease
COVID-19: Corona virus disease
EGFR: Epidermal growth factor receptor
eNose: Electronic nose
FeNO: Exhaled nitric oxide
GOLD: Global Initiative for chronic obstructive lung disease
ILD: Interstitial lung disease
IPF: Idiopathic pulmonary fibrosis
MPM: Malignant pleural mesothelioma
NSCLC: Non-small cell lung cancer
OCS: Oral corticosteroids
OSA: Obstructive sleep apnoea
PCD: Primary ciliary dyskinesia
PD: Programmed death
SCLC: Small cell lung cancer
TB: Tuberculosis
VAP: Ventilator-associated pneumonia
VOCs: Volatile organic compounds
