# Supplementary material for: The smell of lung disease: a review of the current status of electronic nose technology
Source: Respir Res. 2021 Sep 17;22:246. doi: 10.1186/s12931-021-01835-4 (PMC8448171; doi:10.1186/s12931-021-01835-4)
Supplement: Supplementary file 1 — Additional file 1. Sensor technology explained. [file 12931_2021_1835_MOESM1_ESM.docx]

# Additional file 1

## In-depth explanation on sensor technology used in eNoses

In this supplementary text, we provide for each type of sensor a more in-depth explanation how the technology works, the sensor characteristics, advantages and disadvantages, and current use in breath analysis.

### Electrical sensors

A form of electrical sensors often used for gas sensing are conductometric sensors. In conductometric sensors, at the moment, metal oxide semiconductors (MOS) are the most common sensing materials, mainly suitable for gases. Redox reactions between the oxide surface of the sensor and the target gas, induces a reaction on the sensor, resulting in an electronic variation of the oxide surface, which is transduced into a variation of electrical resistance within the sensors. This can be detected by measuring, for example, a change in capacitance, mass or reaction energy [1]. Sensors based on MOS are low cost and have a high sensitivity. However, MOS sensors need to be operated at high temperatures, thus requiring a heating component and leading to high power consumption.

Feasibility studies are conducted for the use of graphene in constructing a MOS sensor [2, 3]. Graphene does not degrade over time and is stable under environmental conditions. Furthermore, graphene is highly sensitive at room temperature, making the use of a heating component unnecessary [4]. However, the fabrication of graphene is an expensive and complex process [4]. Chen et al. constructed an eNose using a metal-ion induced assembly of graphene oxide. They managed to obtain a homogeneous coating of the graphene oxide, creating more excellent gas sensing performances at room temperature [2].

Another used electrical sensor for electronic sensors are (conducting) polymers. Conducting polymer sensors operate based on a change in electrical resistance, caused by the adsorption of an analyte on the sensor surface. Conducting polymers can operate at ambient temperature, thus leading to a lower power consumption than MOS sensors, and are sensitive for an abundance of VOCs. These sensors are, however, easily influenced by humidity and temperature and possess a limited sensor life.

### Gravimetric sensors

Gravimetric sensors operate based on a change in mass, leading to a frequency shift as a sensor response. Gravimetric sensors could be based on either quartz crystal microbalance (QCM), surface acoustic wave (SAW) propagation, or microcantilever. Both the QCM and SAW propagation based sensors use an acoustic wave to detect analytes. The difference is that SAW propagation sensors operate using acoustic waves that travel across the surface of the sensing membrane, while QCM sensors operate based on the piezoelectric effect. For microcantilever based sensors, the presence of a specific analyte causes the cantilever to bend, leading to a frequency shift.

Gravimetric sensors possess a high sensitivity, but also contains a complex circuitry and are sensitive to humidity and temperature.

### Optical sensors

Optical sensors operate based on optical phenomena, for example such as fluorescence and absorbance, caused by the response upon analyte binding. Optical sensors possess a very high sensitivity and specificity, but are in need of complex sensor-array systems, are hardly portable due to breakable optics and components, and are more expensive to use. Therefore, the use of optical sensors in (medical) eNose technology is limited.

# References

1. Liu X, Cheng S, Liu H, Hu S, Zhang D, Ning H. A survey on gas sensing technology. *Sensors (Basel, Switzerland)* 2012: 12(7): 9635-9665.

2. Chen Q, Chen Z, Liu D, He Z, Wu J. Constructing E-Nose Using Metal-Ion Induced Assembly of Graphene Oxide for Diagnosis of Lung Cancer via Exhaled Breath. *ACS Appl Mater Interfaces* 2020.

3. Kovalska E, Lesongeur P, Hogan BT, Baldycheva A. Multi-layer graphene as a selective detector for future lung cancer biosensing platforms. *Nanoscale* 2019: 11(5): 2476-2483.

4. Nag A, Mitra A, Mukhopadhyay SC. Graphene and its sensor-based applications: A review. *Sensors and Actuators A: Physical* 2018: 270: 177-194.

# 
