# Supplementary material for: The smell of lung disease: a review of the current status of electronic nose technology
Source: Respir Res. 2021 Sep 17;22:246. doi: 10.1186/s12931-021-01835-4 (PMC8448171; doi:10.1186/s12931-021-01835-4)
Supplement: Supplementary file 2 — Additional file 2. Search strategy. [file 12931_2021_1835_MOESM2_ESM.docx]

# Additional file 2

## Search strategy

A systematic literature search was performed in three online databases on the 21^st^ of October 2020, as stated below.

Only original articles were included. Articles with no full text available, reviews, abstracts, editorials, congress articles and animal studies were excluded. Moreover, articles were restricted to those investigating eNose technology for clinical purpose; articles only describing techniques as GC or MS, as well as early prototypes of eNose sensor technology were excluded.

*Embase.com*

('electronic nose'/de OR ('mass fragmentography'/de AND ('nose'/de OR 'breath analysis'/exp)) OR 'volatile organic compound'/de OR (eNOSE* OR e-NOSE* OR cyranose* OR spironose* OR ((electronic* OR artificial* OR spectromet* OR GC-MS) NEAR/3 (nose*)) OR volatile-organic-compound* OR VOC):ti,ab,kw) AND ('respiratory tract disease'/exp OR (lung* OR pulmonar* OR respirator*-tract* OR Pneumonolog* OR asthma* OR COPD OR Sarcoidos*):ab,ti,kw) NOT ([Conference Abstract]/lim AND [1800-2017]/py)

*Medline (Ovid)*

(Electronic Nose/ OR (Gas Chromatography-Mass Spectrometry/ AND (Nose/ OR Breath Tests/)) OR volatile organic compound/ OR (eNOSe* OR cyranose* OR spironose* OR ((electronic* OR artificial* OR spectromet* OR GC-MS) ADJ3 (nose*)) OR volatile-organic-compound* OR VOC).ab,ti,kf.) AND (exp Respiratory Tract Diseases/ OR (lung* OR pulmonar* OR respirator*-tract* OR Pneumonolog* OR asthma* OR COPD OR Sarcoidos*).ab,ti,kf.) NOT (news OR congres* OR abstract* OR book* OR chapter* OR dissertation abstract*).pt.

*Cochrane Central*

((eNOSE* OR e-NOSE* OR cyranose* OR spironose* OR ((electronic* OR artificial* OR spectromet* OR GC-MS) NEAR/3 (nose*)) OR volatile-organic-compound* OR VOC):ti,ab,kw) AND ((lung* OR pulmonar* OR respirator* NEXT/1 tract* OR Pneumonolog* OR asthma* OR COPD OR Sarcoidos*):ab,ti,kw)
